# Now in Press

**Published:** 1903-11

**Authors:** 


### Books and Magazines

### Now in Press.

E. B. Treat & Co. announce the early appearance of the following important books:

THE BLUES (Splanchnic Neurasthenia); CAUSES AND CURE.—This form of nervous weakness is so common as to render this volume of more than theoretic interest. By Albert Abrams, M. D., F. R. M. S., 8vo, 230 pages, illustrated, $1.25.

DISEASES OF METABOLISM AND NUTRITION.; Part IV, AUTOINTOXICATION.—By Prof. Dr. Carl von Noorden, Physician-in-Chief to the City Hospital, Frankfort-on-the-Main, and Dr. Mohr. Authorized American edition. Edited by Boardman Reed, M. D. Small 8vo, 80 pages, 50 cents.

We have recently issued the following books:

PLAIN HINTS FOR BUSY MOTHERS.—By Marianna Wheeler, Superintendent of the Babies Hospital, New York. Flexible leatherette, 35 cents.

TREATMENT OF DISEASE BY PHYSICAL METHODS.—Lectures on Electricity, Massage, Baths and Exercise. By Thomas Stretch Dowse, M. D. (abd.), F. R. C. P. (Ed.). Fourth edition. Small 8vo, 454 pages, illustrated, $2.75.

DISEASES OF METABOLISM AND NUTRITION.—A Series of Monographs. By Prof. Dr. Carl von Noorden, Physician-in-Chief to the City Hospital, Frankfort-on-the-Main, and assistants. Authorized American edition. Edited by Boardman Reed, M. D., Philadelphia.

I. Obesity. Small 8vo, 60 pages; cloth, 50 cents. II. Nephritis. Small 8vo, 112 pages; cloth, $1.00. III. Colitis. Small 8vo, 80 pages; cloth, 50 cents.

MEDICAL AND SURGICAL ELECTRICITY.—Including X-Ray, Vibratory Therapeutics, Finsen Light and High Frequency Currents. By A. D. Rockwell, A. M., M. D. New and enlarged edition, royal octavo; illustrated. Half Morocco, $6.00; cloth, $5.00.

